# Lung Sarcomatoid Carcinoma: An Intriguing Case Report of a Rare Malignancy

**DOI:** 10.7759/cureus.39669

**Published:** 2023-05-29

**Authors:** Hajar Charii, Asmae Boudouh, Mohamed Lakhal, Othman Moueqqit, Hatim Kouismi

**Affiliations:** 1 Respiratory Diseases, Mohammed VI University Hospital, Faculty of Medicine and Pharmacy, Mohammed First University, Oujda, MAR; 2 General Medicine, Faculty of Medicine and Pharmacy, Mohammed First University, Oujda, MAR

**Keywords:** case report, chemotherapy, lung sarcomatoide, aggressive, bronchial carcinoma, non-small cell lung carcinoma (nsclc), sarcomatoid carcinomas

## Abstract

Sarcomatoid carcinoma (SC) is a rare primary malignant tumor belonging to the group of non-small cell lung cancer (NSCLC). The diagnosis requires proper tumor sampling to exclude sarcoma, which is the main differential diagnosis. However, the prognosis of these tumors is poor, with a median survival rate lower than other NSCLC cases, mainly due to their aggressiveness and resistance to chemotherapy, especially platinum salts. In this report, we discuss a case of a 62-year-old male patient who presented at admission with hemoptysis and dyspnea. The diagnosis process involved thoracic computed tomography (CT) and fiberoptic bronchoscopy, which revealed tissue thickening at the carina and a mass extending from the lower end of the trachea to the carina, which was confirmed by biopsy. Unfortunately, despite receiving neoadjuvant radiotherapy and having endotracheal prosthesis, the patient succumbed to tumor progression. Our case highlights the aggressive nature of this tumor and underscores the importance of early detection.

## Introduction

Primary sarcomatoid carcinomas (SC) of the lung are extremely rare malignant tumors accounting for less than 1% of non-small cell lung cancer (NSCLC) [1]. Histological examination is crucial for diagnosis, as these tumors encompass a group of poorly differentiated carcinomas exhibiting cells that display a sarcomatous or sarcomatoid morphological appearance, characterized by the presence of giant and/or spindle cells [1]. Clinical and radiological presentations in patients with SC closely resemble those of other NSCLC and are often accompanied by mild symptoms such as fatiguability, chronic cough, chest pain and hemoptysis, which are frequently associated with large tumor size [2-4].

Surgery has a crucial role in localized cases and it is often complemented by adjuvant chemotherapy and radiation therapy. These additional treatments are commonly recommended to specifically target any remaining cancer cells and minimize the risk of recurrence.

## Case presentation

**Figure 1:**
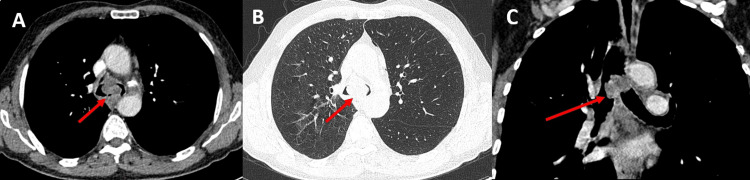
Thoracic CT scan showing tissue thickening (red arrows) that invaded the carina and protruded into the endobronchial region. A: axial section with mediastinal window, B: axial section with parenchymal window, C: coronal section with mediastinal window.

A 62-year-old man with a significant smoking history of 60 pack-years of smoking exposure presented with hemoptysis, dyspnea and weight loss. A computed tomography (CT) scan revealed a mediastinal tumor measuring approximately 37 mm x 28 mm. The tumor had invaded the carina and protruded into the endobronchial region, and was associated with mediastinal lymphadenopathy in the subcarinal region indicating a locally advanced stage classified as T4N2aM0 (Figure 1).

**Figure 2:**
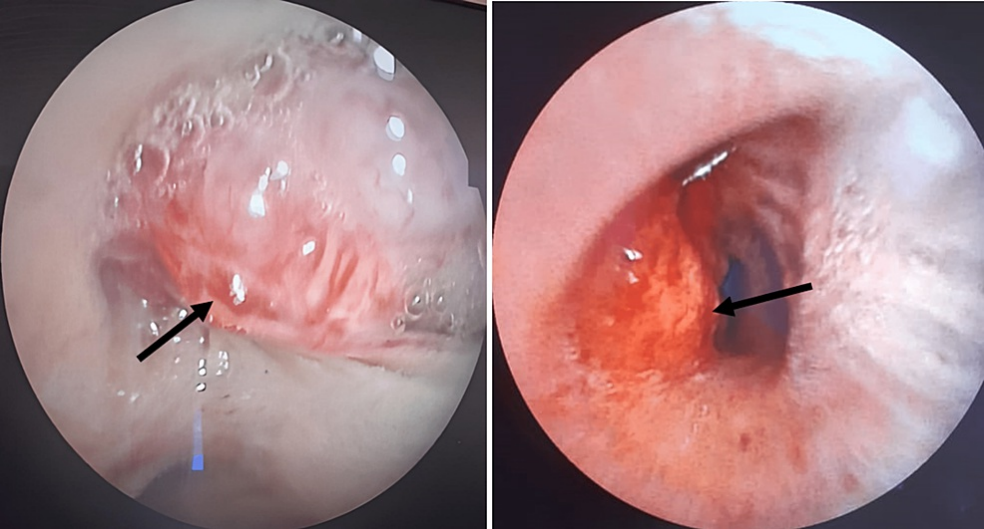
Fiberoptic bronchoscopy reveals a tumoral growth at the lower end of the trachea extending to the carina.

**Figure 3:**
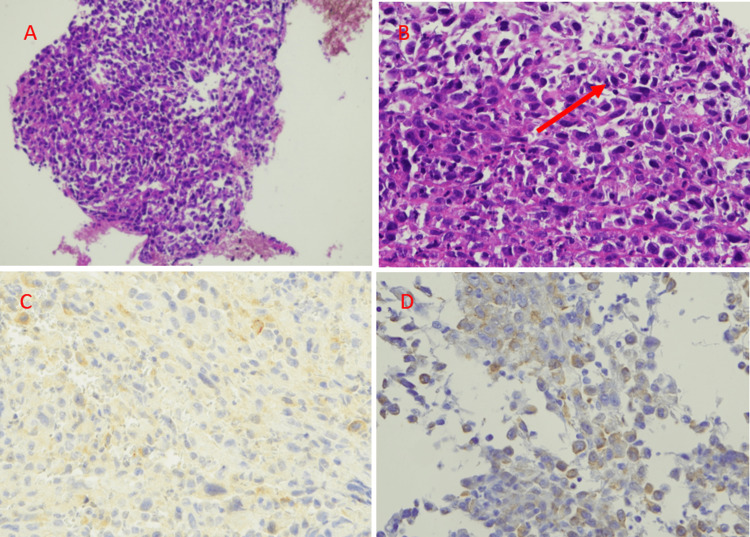
Histopathological examination revealed moderately pleomorphic epithelial cells with oval hyperchromatic nuclei (arrow); stroma consisted of highly pleomorphic oval to spindle-shaped cells along with multinucleated malignant cells (A: x20, B: x40), immunohistochemically stained for cytokeratin 7 (C: x40), CTK (D: x40).

A fiberoptic bronchoscopy confirmed the presence of the mass in the lower end of the trachea, extending to the carina (Figure 2). Following a bronchial biopsy, a diagnosis of SC was established based on the histopathological examination, which revealed epithelial cells displaying moderate pleomorphism, characterized by an oval hyperchromatic nucleus. Additionally, the stroma exhibited pleomorphic oval to spindle-shaped cells, along with the presence of multinucleated malignant cells (Figure 3).

The decision of the multidisciplinary team meeting (MDT) was to place silicone tracheobronchial Y-stent and neoadjuvant radiotherapy (46 gray) for his dyspnea. This led to an initial improvement of his symptoms. After two months, the patient went into hypoxic respiratory failure requiring intubation. The patient did not receive chemotherapy due to a deterioration in their general condition and he passed away after one day of treatment in the intensive care unit.

## Discussion

According to data from the SEER database (Surveillance, Epidemiology, and End Results Program of the National Cancer Institute) [1], SCs, which are a subtype of NSCLC, represent a rare form of tumors, accounting for roughly 0.4%. First documented in 1958 by Nash and Stout in their autopsy study involving five cases, these tumors displayed multinucleated giant cells of different sizes, characterized by hyperchromatic and irregular pleomorphic nuclei, often described as "bizarre" [2].

SCs do not present specific clinical manifestations; however, they exhibit certain characteristics in terms of clinical and radiological presentations compared to other NSCLC. These tumors typically affect male patients with a median age of 60 years, and tobacco use is the main risk factor [5]. Patients often present with respiratory symptoms such as coughing, dyspnea and hemoptysis at the time of diagnosis, and the tumors are frequently large, averaging from 2.1 to 9 cm in size [4]. In our case, the tumor size was approximately 37*28 mm. In two-thirds of cases, the tumor is peripheral, capable of invading the pleura and ribs, and causing pain. Upper lobes are more commonly affected [4]. In one-third of cases, the tumor is centrally located with endobronchial, mediastinal or vascular extension, as seen in our case, which resulted in hemoptysis in 20% of cases [3].

Histological diagnosis can be challenging as it is based on the analysis of surgical specimens, although cytological characteristics can also be considered [3]. As a result, establishing the diagnosis of SC on small biopsy fragments is often difficult. The pathologist's ability to diagnose this voluminous and heterogeneous tumor, which is frequently necrotic, relies on obtaining an ample number of samples from preserved areas of necrosis at the tumor periphery to visualize its different components. On average, 26 blocks are examined to achieve a diagnosis [3]. In our patient's case, the histological diagnosis was established based on a small bronchial biopsy. Immunohistochemistry is utilized to confirm the presence of a carcinomatous proliferation predominantly composed of fusiform or giant cells by detecting the positivity of pancytokeratins. Pancytokeratin AE1/AE3 is considered the most sensitive marker for identifying the presence of an epithelial component, although it does not include CK18, which can sometimes be expressed independently [6]. Cytokeratin 7 and TTF-1 are frequently expressed in the epithelial component of adenocarcinomas and may suggest a primary pulmonary origin.

The therapeutic management of SCs primarily relies on the same principles as NSCLC, which mainly involves surgical approaches. However, the risk of tumor recurrence following surgical resection is high, primarily due to the sarcomatous component [7]. The prognosis for SC is unfavorable, with a survival rate of less than 10% at two years [5]. Adjuvant therapies such as chemotherapy and radiotherapy are sometimes employed but have limited effectiveness [5]. Platinum-based chemotherapy as a first-line treatment has yielded disappointing results, with a progression rate of 69%. In a study involving 178 stage IIIb-IV NSCLC patients undergoing chemotherapy, the sarcomatoid subtype was the only factor predicting resistance to first-line platinum-based chemotherapy, with a multivariate analysis showing a 7.5-fold increased risk of developing resistance to first-line platinum-based chemotherapy [8]. The efficacy of targeted therapies in this context remains unknown, while immunotherapy is emerging as a promising therapeutic option.

Complete tumor resection stands as a pivotal factor in the management of SC, particularly in cases of localized tumors at an early stage. The decision regarding surgical treatment eligibility could be guided by the patient's Eastern Cooperative Oncology Group (ECOG) score assessment [9].To optimize treatment strategies for SC, it is crucial to tailor therapy based on gene detection results and adherence to established indications [9]. In the realm of anaplastic lymphoma kinase inhibitors, second-generation options like ceritinib, alectinib, and brigatinib, as well as third-generation alternatives such as lorlatinib, exhibit potential in extending the survival time of SC patients with anaplastic lymphoma kinase rearrangement when compared to their first-generation counterparts [9]. Furthermore, the early utilization of antiangiogenic therapy and immune-checkpoint inhibitors holds promise in maximizing treatment responses and significantly improving the quality of life for individuals afflicted with SC. These therapeutic approaches serve to expand the treatment landscape not only for SC but also for other rare diseases [9]. Moreover, antiangiogenic targeted drugs like apatinib and monoclonal antibodies such as bevacizumab can also be considered for patients who have developed resistance to crizotinib. The overexpression of programmed cell-death protein-1/programmed cell-death ligand-1 (PD-L1/PD1) and the presence of high vascular invasiveness provide a solid rationale for exploring the use of immune-checkpoint inhibitors [9]. Considering future prospects, targeting MET mutation and investigating the potential of therapies like TP53 mutation reversal drugs, CDK 4/6 inhibitors, and MEK inhibitors hold promise as potent treatment options. These endeavors aim to further enhance our understanding and pave the way for more effective interventions in the management of SC [9].

## Conclusions

SCs are rare tumors with an unfavorable prognosis, presenting particular morphological and molecular characteristics. They are associated with a high rate of progression under first-line platinum-based chemotherapy. It is necessary to recognize this pathological entity and to characterize it more precisely at the molecular level in order to propose a specific therapeutic approach.
